# Could serum total cortisol level at admission predict mortality due to coronavirus disease 2019 in the intensive care unit? A prospective study

**DOI:** 10.1590/1516-3180.2020.0722.R1.2302021

**Published:** 2021-06-25

**Authors:** Mehmet Güven, Hamza Gültekin

**Affiliations:** I MD. Endocrinologist, Department of Endocrinology and Metabolism, Şırnak State Hospital, Şırnak, Turkey.; II MD. Physician, Department of Intensive Care, Şırnak State Hospital, Şırnak, Turkey.

**Keywords:** COVID-19, Intensive care units, Mortality, Coronavirus disease 2019, Intensive care center, Cortisol

## Abstract

**BACKGROUND:**

Critical diseases usually cause hypercortisolemia via activation of the hypothalamic-pituitary-adrenal axis.

**OBJECTIVES:**

To investigate the relationship between serum total cortisol level and mortality among coronavirus disease 2019 (COVID-19) patients in the intensive care unit (ICU), at the time of their admission.

**DESIGN AND SETTING:**

Prospective study developed in a pandemic hospital in the city of Şırnak, Turkey.

**METHODS:**

We compared the serum total cortisol levels of 285 patients (141 COVID-19-negative patients and 144 COVID-19-positive patients) followed up in the ICU.

**RESULTS:**

The median cortisol level of COVID-19-positive patients was higher than that of COVID-19 negative patients (21.84 μg/dl versus 16.47 μg/dl; P < 0.001). In multivariate logistic regression analysis, mortality was associated with higher cortisol level (odds ratio: 1.20; 95% confidence interval: 1.08-1.35; P = 0.001). The cortisol cutoff point was 31 μg/dl (855 nmol/l) for predicting mortality among COVID-19-positive patients (area under the curve 0.932; sensitivity 59%; and specificity 95%). Among the COVID-19 positive patients with cortisol level ≤ 31 μg/dl (79%; 114 patients), the median survival was higher than among those with cortisol level > 31 μg/dl (21%; 30 patients) (32 days versus 19 days; log-rank test P < 0.001).

**CONCLUSION:**

Very high cortisol levels are associated with severe illness and increased risk of death, among COVID-19 patients in the ICU.

## INTRODUCTION

The new virus infection that first appeared in Wuhan, China, at the end of December 2019 spread to most countries across the world, causing a global pandemic. In February 2020, the World Health Organization (WHO) named the virus severe acute respiratory syndrome-coronavirus-2 (SARS-CoV-2) and the associated infectious disease, novel coronavirus 2019 (COVID-19).^1^

Acute stress from critical illness increases serum cortisol levels via activation of the hypothalamic-pituitary-adrenal (HPA) axis.^2^^,^^3^^,^^4^ This is the body’s adaptive survival mechanism. SARS-CoV-2 enters pneumocytes using the host’s angiotensin-converting enzyme 2 (ACE2) receptors. This enzyme is also found in the arterial and venous endothelial cells of several endocrine organs, including the adrenals.^5^^,^^6^^,^^7^ Therefore, the HPA axis may be affected during the course of SARS-CoV-2 infection.

The relationship between cortisol release caused by COVID-19 and mortality in the intensive care unit (ICU) is yet to be adequately studied.

## OBJECTIVE

The present prospective study was designed with the aim of investigating the relationship between mortality and serum total cortisol levels, measured in a sample collected from COVID-19 patients on the first morning of admission to the ICU of the hospital.

## METHODS

This prospective study was conducted among 285 patients hospitalized in the ICU of the Şırnak State Hospital (a pandemic hospital in Turkey), over the period from March 15, 2020, to August 15, 2020. Group 1 included 141 COVID-19-negative patients, and group 2 comprised 144 COVID-19-positive patients.

The study protocol was approved by the Clinical Research Ethics Committee of the Health Sciences University Gazi Yaşargil Training and Research Hospital (Diyarbakır, Turkey) (September 11, 2020; number: 546).

COVID-19 positivity was based on positive results from real-time reverse transcriptase polymerase chain reaction (RT-PCR) tests on nasopharyngeal and oropharyngeal swabs, or on the presence of strong clinical and radiological signs, even in cases in which the swab result was negative.

Samples were collected in the morning, between 7 AM and 8 AM, of the day when the patient was admitted to the ICU. These were used to evaluate the complete blood count, creatinine level, aspartate aminotransferase (AST) level, alanine aminotransferase (ALT) level, albumin level, C-reactive protein (CRP) level, D-dimer level and serum total cortisol level.

Cortisol levels were analyzed using the Cobas 6000 analyzer (Roche Diagnostics, Mannheim, Germany). The reference range for serum total cortisol measured using this device was 0.018-63.4 μg/dl.

### Exclusion criteria

Patients with a history of previously known pituitary disorder, adrenal gland disorder, use of corticosteroids (inhaler, topical, oral or parenteral) or use of other drugs that could have disrupted the HPA axis during the previous 3 months, and those on mechanical ventilation at the time of sample collection were excluded from the study. All other patients aged > 18 years were enrolled.

### Statistical analyses

The data were analyzed statistically using the Statistical Package for the Social Sciences (SPSS) for Windows software, version 22 (IBM, Chicago, Illinois, United States). Continuous variables were presented as medians with the corresponding interquartile range (IQR), and categorical variables were presented as frequencies and percentages. The chi-square test was used to analyze the categorical variables. Comparisons were made using the Mann-Whitney U test, Kruskal-Wallis test and one-way analysis of variance (ANOVA) test. Spearman’s correlation test was used to examine the relationships between pairs of variables. Univariate and multivariate logistic regression analyses were used to detect predictors of mortality. Receiver operating characteristic (ROC) analysis and the area under the curve (AUC) were used to examine the serum total cortisol levels with regard to predicting patient mortality. Sensitivity and specificity values were calculated, and the optimal cutoff levels for serum cortisol were defined. Kaplan-Meier survival curves and the log-rank test were used to examine overall survival. P < 0.05 was considered to indicate statistical significance.

## RESULTS

In group 1 (COVID-19-negative patients), 63% (89/141) were male patients, while in group 2 (COVID-19-positive patients), 61% (88/144) were men. The predominance of male patients in the groups was noteworthy. The median age of the group 1 patients was 61 years (IQR, 50.5-73.5 years), while that of group 2 was 63.5 years (IQR, 52.25-69 years).

While the proportion of the patients who died was 12% (n = 17) in group 1, it was 30.5% in group 2 (n = 44; P < 0.001). The median length of stay in the ICU was 7 days (IQR, 5-12 days) in group 1, while it was 17 days (IQR, 11-24.5 days) in group 2 (P < 0.001). The CRP level was significantly higher in group 2 than in group 1 (P < 0.001). The median cortisol level was 16.47 μg/dl (IQR, 13.73-19.13 μg/dl) in group 1 and 21.84 μg/dl (18.22-30.11 μg/dl) in group 2 (P < 0.001). The demographic, clinical and laboratory parameters of the groups are shown in Table 1.

**Table 1.** Demographic, clinical and laboratory parameters of the groups

Parameters		Group 1 (n = 141) COVID-19-negative	Group 2 (n = 144) COVID-19-positive	P-value
Age, years		61 (50.5-73.5)	63.5 (52.25-69)	0.852
Age (stratified)	< 45	17 (12.1%)	24 (16.7%)	0.268
	45-64	63 (44.7%)	52 (36.1%)	0.14
	65-74	34 (24.1%)	53 (36.8%)	0.02
	≥ 75	27 (19.1%)	15 (10.4%)	0.038
Sex	Male	89 (63%)	88 (61%)	0.727
	Female	52 (37%)	56 (39%)	0.727
Length of stay, days		7 (5-12)	17 (11-24.5)	< 0.001
Death		17 (12%)	44 (30.5%)	< 0.001
Cortisol, μg/dl		16.47 (13.73-19.13)	21.84 (18.22-30.11)	< 0.001
CRP, mg/dl		12.14 (9.27-14.24)	19.7 (15.87-31.29)	< 0.001
D-dimer, ug/l		680 (482-1050)	1250 (1055-2400)	< 0.001
Creatine, mg/dl		0.92 (0.79-1.25)	1.04 (0.81-1.78)	0.025
AST, U/l		31 (19.5-42)	36.5 (25-56)	0.001
ALT, U/l		28 (14.5-39)	26 (17-46.5)	0.167
Albumin, g/dl		2.91 (2.46-3.59)	2.7 (2.38-3.05)	< 0.001
Neutrophil-to-leukocyte (N:L) ratio		5.62 (3.26-10.33)	8.83 (4.00-13.87)	0.002

CRP = C-reactive protein; AST = aspartate aminotransferase; ALT = alanine aminotransferase. Categorical data shown as number (percentage). Non-normally distributed continuous variables displayed as median (interquartile range [IQR]). P < 0.05: significant (shown in bold).

The COVID-19-positive group was divided into subgroups, as survivors (n = 100) and non-survivors (n = 44). The median age of the survivors was 64 years (IQR, 56.25-68 years), while that of the non-survivors was 61 years (IQR, 43.5-69 years). The majority of the patients in both groups were men. The CRP level was 18.76 mg/dl (IQR, 14.69-28.84 mg/dl) in the survivor group and 25.81 mg/dl (17.18-33.1 mg/dl) in the non-survivor group (P < 0.05). The demographic, clinical and laboratory results of the survivor and non-survivor COVID-19 patients are shown in Table 2.

**Table 2.** Characteristics of COVID-19-positive patients

Parameters		Survivors (n = 100)	Non-survivors (n = 44)
Age, years		64 (56.25-68)	61 (43.5-69)
Age (stratified)	< 45	13 (13%)	11 (25%)
	45-64	38 (38%)	14 (32%)
	65-74	41 (41%)	12 (27%)
	≥ 75	8 (8%)	7 (16%)
Sex	Male	55 (55%)	33 (75%)
	Female	45 (45%)	11 (25%)^*^
Length of stay, days		17.5 (11.25-22)	16.5 (9-25)
CRP, mg/dl		18.76 (14.69-28.84)	25.81 (17.18-33.1)^**^
D-dimer, ug/l		1360 (1007-2460)	1212 (1062-1871)
Creatine, mg/dl		1.03 (0.76-1.69)	1.29 (0.93-2.08)
AST, U/l		34 (24.25-50.75)	40.5 (26-64.25)
ALT, U/l		26.5 (18-45)	20.5 (14.25-50.25)
Albumin, g/dl		2.74 (2.39-3.02)	2.61 (1.77-3.11)
Neutrophil-to-leukocyte (N:L) ratio		8.97 (3.97-13.29)	8.64 (4.08-15.68)

CRP = C-reactive protein; AST = aspartate aminotransferase; ALT = alanine aminotransferase. Categorical data shown as number (percentage). Non-normally distributed continuous variables displayed as median (interquartile range [IQR]). P < 0.05: significant; ^*^P < 0.05 (chi-square test; ^**^P < 0.05 (Mann-Whitney test).

**Figure 1.**
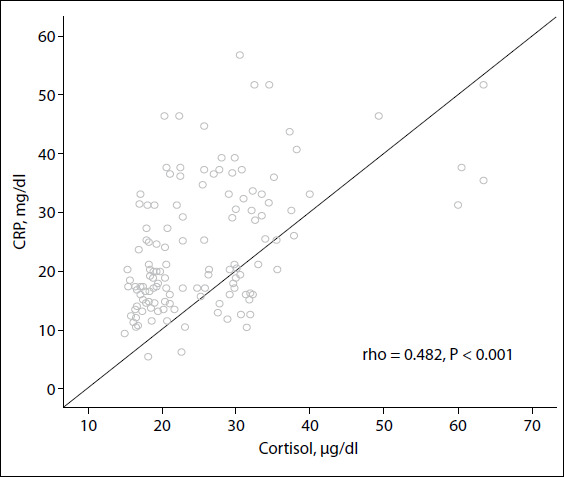
Positive correlation of cortisol with C-reactive protein (CRP) levels in the COVID-19-positive group in the intensive care unit (Spearman’s correlation).

**Figure 2.**
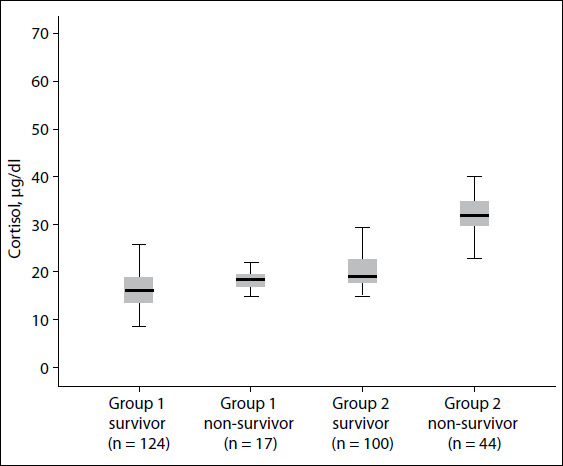
Boxplot of cortisol distribution of the groups (Group 1, COVID-19-negative; Group 2, COVID-19-positive). The thick line in the middle of each box is the median. The top and bottom lines of each box indicate the first and third quartiles.

In the COVID-19-positive group, the cortisol and CRP levels were positively correlated (rho: 0.482; P < 0.001) (Figure 1). The patients in the groups were divided into subgroups as survivors and non-survivors, and their cortisol levels were compared. The median cortisol level of group 1 survivors (n = 123) was 16.3 μg/dl, while that of group 1 non-survivors (n = 17) was 18.4 μg/dl. Group 2 survivors (n = 100) had a median cortisol level of 19.05 μg/dl, while group 2 non-survivors (n = 44) had a median cortisol level of 31.8 μg/dl. The box plot of the cortisol distribution of the subgroups is presented in Figure 2. Group 2 non-survivors had the highest cortisol level, according to multiple comparisons of the serum cortisol levels of the subgroups using the Kruskal-Wallis and one-way ANOVA tests (P < 0.001) (Table 3).

**Table 3.** Comparison of the median cortisol levels of survivors and non-survivors

Groups	Median cortisol level	Interquartile range	Groups	P-value
Group 1 survivor (n = 123)	16.3	13.45-18.92	Group 1 non-survivor	0.624
			Group 2 survivor	< 0.001
			Group 2 non-survivor	< 0.001
Group 1 non-survivor (n = 17)	18.4	16.11-19.56	Group 1 survivor	0.624
			Group 2 survivor	0.006
			Group 2 non-survivor	< 0.001
Group 2 survivor (n = 100)	19.05	17.76-22.54	Group 1 survivor	< 0.001
			Group 1 non-survivor	0.006
			Group 2 non-survivor	< 0.001
Group 2 non-survivor (n = 44)	31.8	29.64-34.92	Group 1 survivor	< 0.001
			Group 1 non-survivor	< 0.001
			Group 2 survivor	< 0.001

Group 1 = COVID-19 negative patients, Group 2 = COVID-19 positive patients Comparisons between groups were made using the Kruskal-Wallis test and one-way analysis of variance. Multiple comparisons between groups were made using the post-hoc Tamhane T2 test. P < 0.05 = significant (shown in bold).

Univariate logistic regression analysis on the factors that influenced mortality among the COVID-19-positive patients in the ICU, including male sex, cortisol level, CRP level and albumin level, showed significant results (Table 4). Multivariate logistic regression analysis on the factors affecting mortality showed that the cortisol level (odds ratio: 1.20; 95% CI: 1.08-1.35; P = 0.001) was a significant factor (Table 5).

**Table 4.** Univariate logistic regression analysis on clinical parameters related to in-hospital mortality among patients diagnosed with COVID-19 in the intensive care unit

Parameter	B	Wald	P value	Odds ratio	95% CI	
					Lower	Upper
Sex (male)	-0.898	4.989	0.026	0.407	0.185	0.896
Age, years	-0.013	0.756	0.385	0.987	0.958	1.017
Length of stay, days	0.001	0.004	0.952	1.001	0.961	1.043
Cortisol, μg/dl	0.221	32.811	0.000	1.247	1.156	1.345
CRP, mg/dl	0.039	5.228	0.022	1.040	1.006	1.075
D-dimer, ug/l	0.000	1.219	0.270	1.000	0.999	1.000
Creatine, mg/dl	0.118	0.760	0.383	1.125	0.863	1.465
AST, U/l	0.001	0.750	0.386	1.001	0.998	1.005
ALT, U/l	0.001	0.100	0.751	1.001	0.996	1.006
Albumin, g/dl	-0.622	4.171	0.041	0.537	0.296	0.975
N:L ratio	-0.021	1.938	0.164	0.979	0.950	1.009

CI = confidence interval; CRP = C-reactive protein; AST = aspartate aminotransferase, ALT = alanine aminotransferase; N:L ratio: neutrophil-to-leukocyte ratio. P < 0.05: significant (shown in bold).

**Table 5.** Multivariate logistic regression analysis on clinical parameters related to in-hospital mortality among patients diagnosed with COVID-19 in the intensive care unit

Parameters	B	Wald	P-value	Odds ratio	95% CI	
					Lower	Upper
Age, years	-0.017	0.610	0.435	0.983	0.940	1.027
Sex (male)	-0.240	0.172	0.678	0.787	0.253	2.447
Length of stay, days	-0.022	0.438	0.508	0.979	0.918	1.043
Cortisol, μg/dl	0.190	11.397	0.001	1.209	1.083	1.350
CRP, mg/dl	-0.032	1.104	0.293	0.968	0.912	1.028
D-dimer, ug/l	0.000	1.219	0.270	1.000	0.999	1.000
Creatine, mg/dl	-0.003	0.000	0.989	0.997	0.644	1.543
AST, U/l	0.001	0.033	0.856	1.001	0.987	1.016
ALT, U/l	-0.008	0.364	0.546	0.992	0.967	1.018
Albumin, g/dl	-0.489	1.154	0.283	0.613	0.251	1.497
N:L ratio	-0.019	0.693	0.405	0.981	0.938	1.026

CI = confidence interval; CRP = C-reactive protein; AST = aspartate aminotransferase; ALT = alanine aminotransferase; N:L ratio: neutrophil-to-leukocyte ratio. P < 0.05: significant (shown in bold).

**Figure 3.**
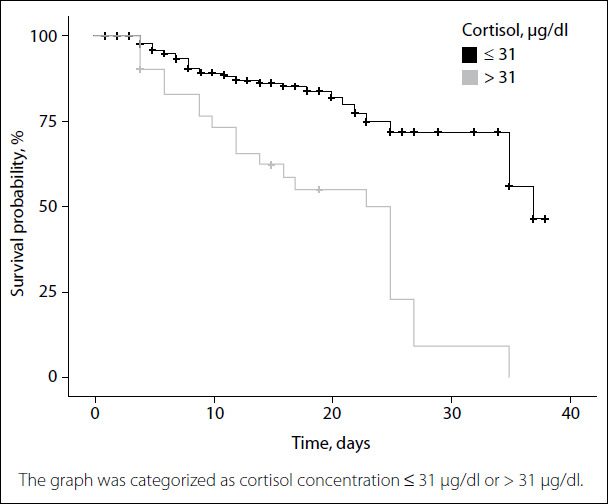
Kaplan-Meier plot of survival probability over time.

ROC curve analysis on cortisol was performed to predict mortality among COVID-19-positive patients. The cortisol cutoff point was found to be 31 μg/dl (855 nmol/l) (AUC 0.932; sensitivity 59% and specificity 95%). The positive predictive value was 57% and the negative predictive value was 95%, for the cortisol level 31 μg/dl (Table 6). With the cortisol level set at 31 μg/dl as the cutoff point, the median survival of those with cortisol level ≤ 31 μg/dl in the Kaplan-Meier life analysis was 32 days (114 patients [79%]; 95% CI: 24 to undetermined), and the median survival of those with cortisol level > 31 μg/dl was 19 days (30 patients [21%]; 95% CI: 20.69-29.31) (log-rank test, P < 0.001) (Figure 3).

**Table 6.** Receiver operating characteristic curve analysis on cortisol to predict mortality among COVID-19-positive patients

Parameter	Cutoff point	AUC	Sensitivity	Specificity	PPV	NPV
Cortisol	31 μg/dl (855 nmol/l)	0.932	59%	95%	57%	95%

AUC = area under the curve; PPV = positive predictive value; NPV = negative predictive value.

## DISCUSSION

Despite all the support and treatments for COVID-19, the mortality rates among patients admitted to ICUs remain high across the world. In a study on 3,988 patients in Italy, the mortality rate in the ICU was 48.7%.^8^ A study on 3,001 patients admitted to ICUs in the United Kingdom found a mortality rate of 31 %.^9^ In a study on patients in 178 ICUs in Turkey, the mortality rate was 55.6%.^10^ In our study, the mortality rate was 12% among COVID-19-negative patients who were admitted to the ICU, while it was 30.5% among COVID-19-positive patients. Development of acute, progressive respiratory failure caused by COVID-19 infection over a short time is associated with a high risk of mortality.

Similar to what was observed in previous studies, ^11^^,^^12^^,^^13^ our study showed that male sex, high CRP level and low albumin level were associated with high risk of mortality. Although the COVID-19-negative and COVID-19-positive patients did not differ in terms of age, there were more patients aged > 65 years in the COVID-19-positive group. Furthermore, the levels of inflammatory markers (CRP, d-dimer and neutrophil-to-leukocyte ratio), creatinine level and AST level were higher and albumin levels were lower in the COVID-19-positive group than in the COVID-19-negative group.

In stress-free individuals, cortisol is secreted in a daily pattern, with levels peaking in the early morning and dropping to their lowest level in the late evening. Any kind of acute illness or trauma leads to changes in daily cortisol secretion,^14^^,^^15^ often accompanied by hypercortisolemia in proportion to disease severity, in cases of critical illness that causes severe acute physical stress.^16^^,^^17^^,^^18^ The increase in cortisol release owing to acute stress in the ICU is an adaptive mechanism of the body that triggers regulation of cardiovascular, immune and metabolic functions. An appropriate response from the HPA axis to the severe stress of critical illness is essential for survival because both very high cortisol responses and low responses (relative adrenal insufficiency) have been associated with higher mortality rates.^19^^,^^20^^,^^21^^,^^21^ In our study, the total serum cortisol level on the first morning of hospitalization in the ICU was significantly higher in the COVID-19-positive patients than in the COVID-19-negative patients (21.84 versus 16.47 μg/dl; P < 0.001).

This may be attributable to the fact that COVID-19-positive patients had more severe disease and higher CRP level. Specific cytokines, such as CRP, which presents elevated concentrations in critical illness, activate the HPA axis and modulate the activity of 11β-hydroxysteroid dehydrogenase (an enzyme involved in steroid hormone physiology) and the number, affinity, or both, of glucocorticoid receptors.^19^^,^^22^ Moreover, the spread of COVID-19 infection has been termed a fatal pandemic by news channels and newspapers; therefore, it is likely to cause fear of death among COVID-19-positive patients, thus leading to greater severity of acute stress and higher stimulation of cortisol release.

A comparison of the baseline cortisol levels of 403 COVID-19-positive patients and 132 COVID-19-negative patients by Tan et al. showed significantly higher cortisol levels in COVID-19-positive patients than in COVID-19-negative patients (619 nmol/l versus 519 nmol/l; P < 0.0001).^23^ The COVID-19-positive group in that previous study had higher CRP levels, similar to those of our study; thus, we can conclude that cortisol elevation may be related to disease severity. In another study on 62 patients with severe sepsis and 63 with septic shock who were admitted to an ICU, the baseline total cortisol levels were 728 ± 386 nmol/l and 793 ± 439 nmol/l, respectively. Non-survivors had higher calculated total cortisol concentrations (980 ± 458 nmol/l) than the survivors (704 ± 383 nmol/l).^24^

The lowest median cortisol level was in group 1 survivors, while the group 2 non-survivors had the highest cortisol level in the multiple-group comparisons that were performed by dividing the study subjects into subgroups of survivors and non-survivors. In the multivariate logistic regression analysis, in which we examined the effect of clinical parameters on mortality among COVID-19-positive patients, only the cortisol level was significant.

The cortisol cutoff point was 31 μg/dl (855 nmol/l) in the ROC curve analysis that was performed to predict mortality among the COVID-19-positive patients (sensitivity 59% and specificity 95%). It is challenging to predict, on the day of ICU admission, which patients are likely to die. In fact, it is difficult for clinicians to select patients for whom more time should be devoted, given the limited time and resources. In such cases, the serum total cortisol level could facilitate and guide the decision-making process for clinicians.

Through using the cutoff point of 31 μg/dl for our patients’ cortisol level, the median length of survival for cortisol levels ≤ 31 μg/dl was 32 days, while that for cortisol levels > 31 μg/dl was 19 days. We found that this significant value was an important marker that could be used to estimate mortality among COVID-19-patients admitted to the ICU.

The present study had certain limitations. First, we only performed analysis using a single baseline cortisol level that was measured on the first morning of admission to the ICU due to COVID-19. In our ICU, corticosteroid treatment is frequently applied from the first day of admission; therefore, measuring the serum cortisol levels under corticosteroid treatment may have provided inaccurate results. Second, we did not measure the level of adrenocorticotropic hormone (ACTH), cortisol-binding globulin or free cortisol. Therefore, it was not possible to comprehensively evaluate the effects of COVID-19 on the HPA axis. Third, no ACTH stimulation test (synacthen test) was performed on the patients before starting this study. Therefore, an unknown state of adrenal insufficiency may have been overlooked. Lastly, this study was conducted in a single center. Larger, multicenter studies are required, in order to obtain more conclusive evidence.

## CONCLUSIONS

Elevated cortisol level is an independent biomarker that enables prediction of adverse outcomes and mortality among COVID-19-positive patients admitted to the ICU. The ability to predict which patients in the ICU may deteriorate faster will help clinicians to allocate resources appropriately and raise the standard of patient care. Furthermore, we can consider a patient’s cortisol levels while making a decision regarding the treatment approach.

## References

[1] World Health Organization Director-General’s remarks at the media briefing on 2019-nCoV on 11 February 2020.

[2] Téblick A, Peeters B, Langouche L, Van den Berghe G. (2019). Adrenal function and dysfunction in critically ill patients. Nat Rev Endocrinol.

[3] Widmer IE, Puder JJ, König C (2005). Cortisol response in relation to the severity of stress and illness. J Clin Endocrinol Metab.

[4] Marik PE, Zaloga GP. (2002). Adrenal insufficiency in the critically ill: a new look at an old problem. Chest.

[5] Hoffmann M, Kleine-Weber H, Schroeder S (2020). SARS-CoV-2 Cell Entry Depends on ACE2 and TMPRSS2 and Is Blocked by a Clinically Proven Protease Inhibitor. Cell.

[6] Yan R, Zhang Y, Li Y (2020). Structural basis for the recognition of SARS-CoV-2 by full-length human ACE2. Science.

[7] Li X, Geng M, Peng Y, Meng L, Lu S. (2020). Molecular immune pathogenesis and diagnosis of COVID-19. J Pharm Anal.

[8] Grasselli G, Greco M, Zanella A (2020). Risk Factors Associated With Mortality Among Patients With COVID-19 in Intensive Care Units in Lombardy, Italy. JAMA Intern Med.

[9] Docherty AB, Harrison EM, Green CA (2020). Features of 20 133 UK patients in hospital with covid-19 using the ISARIC WHO Clinical Characterisation Protocol: prospective observational cohort study. BMJ.

[10] Solmaz I, Özçaylak S, Alakuş ÖF (2020). Risk factors affecting ICU admission in COVID-19 patients; Could air temperature be an effective factor?. Int J Clin Pract.

[11] Güner R, Hasanoğlu İ, Kayaaslan B (2020). COVID-19 experience of the major pandemic response center in the capital: results of the pandemic’s first month in Turkey. Turk J Med Sci.

[12] Feng Y, Ling Y, Bai T (2020). COVID-19 with Different Severities: A Multicenter Study of Clinical Features. Am J Respir Crit Care Med.

[13] Richardson S, Hirsch JS, Narasimhan M (2020). Presenting Characteristics, Comorbidities, and Outcomes Among 5700 Patients Hospitalized With COVID-19 in the New York City Area. JAMA.

[14] Boonen E, Vervenne H, Meersseman P (2013). Reduced cortisol metabolism during critical illness. N Engl J Med.

[15] Mesotten D, Vanhorebeek I, Van den Berghe G. (2008). The altered adrenal axis and treatment with glucocorticoids during critical illness. Nat Clin Pract Endocrinol Metab.

[16] Cooper MS, Stewart PM. (2003). Corticosteroid insufficiency in acutely ill patients. N Engl J Med.

[17] Rivier C, Vale W. (1983). Modulation of stress-induced ACTH release by corticotropin-releasing factor, catecholamines and vasopressin. Nature.

[18] Rook GA. (1999). Glucocorticoids and immune function. Baillieres Best Pract Res Clin Endocrinol Metab.

[19] Cooper MS, Bujalska I, Rabbitt E (2001). Modulation of 11beta-hydroxysteroid dehydrogenase isozymes by proinflammatory cytokines in osteoblasts: an autocrine switch from glucocorticoid inactivation to activation. J Bone Miner Res.

[20] Vermes I, Beishuizen A. (2001). The hypothalamic-pituitary-adrenal response to critical illness. Best Pract Res Clin Endocrinol Metab.

[21] Rothwell PM, Lawler PG. (1995). Prediction of outcome in intensive care patients using endocrine parameters. Crit Care Med.

[22] Marx C, Petros S, Bornstein SR (2003). Adrenocortical hormones in survivors and nonsurvivors of severe sepsis: diverse time course of dehydroepiandrosterone, dehydroepiandrosterone sulfate, and cortisol. Crit Care Med.

[23] Tan T, Khoo B, Mills EG (2020). Association between high serum total cortisol concentrations and mortality from COVID-19. Lancet Diabetes Endocrinol.

[24] Bendel S, Karlsson S, Pettilä V (2008). Free cortisol in sepsis and septic shock. Anesth Analg.

