# Erratum

**DOI:** 10.1590/1516-3180.2020.0722.R1.2302021erratum

**Published:** 2022-06-27

In the manuscript titled “Could serum total cortisol level at admission predict mortality due to coronavirus disease 2019 in the intensive care unit? A prospective study”, DOI number 10.1590/1516-3180.2020.0722.R1.2302021, published in the Sao Paulo Medical Journal, volume 139, issue number 4, pages 398-404, on page 399:

**Where it read:**

“The study protocol was approved by the Clinical Research Ethics Committee of the Health Sciences University Gazi Yaşargil Training and Research Hospital (Diyarbakır, Turkey) (September 11, 2020; number: 546).”

**It should read:**

“The study protocol was approved by the Clinical Research Ethics Committee of the Health Sciences University Gazi Yaşargil Training and Research Hospital (Diyarbakır, Turkey) (March 11, 2020; number: 38).”

